# Retroviruses and the Third Synapse

**DOI:** 10.3390/v2041008

**Published:** 2010-04-15

**Authors:** Quentin J. Sattentau

**Affiliations:** The Sir William Dunn School of Pathology, The University of Oxford, South Parks Road, Oxford OX13RE, UK; E-Mail: quentin.sattentau@path.ox.ac.uk; Tel.: +44 1865 275511; Fax; +44 1865 275515

The direct movement of viruses between contacting cells as a mode of dissemination distinct from the release of cell-free virions was hinted at in pioneering experiments first reported almost eighty years ago [1], and confirmed and extended 30 years later [2,3]. This early work was carried out using the tools of the time in the absence of the modern cell biological, immunological and virological techniques available today. As such, although many of the basic concepts were established for cell-to-cell spread prior to the discovery of retroviruses, descriptions of the molecular and cellular mechanisms underlying this phenomenon were lacking. Papers from two decades ago revealed that HIV-1 could spread between cultured lymphocytes by cell-to-cell spread [4], proposed that this mechanism of dissemination was substantially more efficient than diffusion-limited spread of cell-free virions [5,6], and suggested that this might be a mechanism of evasion from antibody neutralization [4].

Investigation of the cell-to-cell spread of viruses, and particularly retroviruses, has seen a renaissance in the past five years with the discovery of a multi-molecular structure termed the virological, or infectious, synapse [7–10]. The definition of this structure was to a great extent based upon the paradigm established by two other well-established synaptic junctions, neural and immunological synapses [11], and the virological synapse shares features of these synapses. Foremost amongst these shared features are the relatively stable adhesive junction formed between the pre-synaptic (virus infected donor) cell and the post-synaptic (receptor-expressing target) cell, and the cytoskeleton-dependent directed release of intercellular information, which in the case of the virological synapse is infectious material in the form of virions. Thus the virological synapse becomes a ‘third synapse’, distinct from the neural and immunological synapses in that it transfers ‘pathogenic information’ between cells. Although first described for retroviruses, other viruses can use virological synapses for spread between immune cells [12], and the list will no doubt grow longer.

Although we do not yet have direct evidence supporting a role for retroviral cell-to-cell spread *in vivo*, its importance seems certain. HTLV-1 is almost non-infectious *in vitro* in a cell-free form, strongly implying that the predominant means of spread *in vitro* and *in vivo* is cell-to-cell, and helping to explain *in vivo* viral tropism [13]. In the early stages of HIV-1 infection the virus infects and kills CD4^+^ T cells so rapidly that the comparatively slow dissemination by cell-free virus is unlikely to account for this [14]. Moreover, HIV-1 preferentially targets CD4^+^ T cells with T cell receptors specific for itself, implying that the virus is able to infect such cells across immunological synapses [15]. Finally, the focal distribution of SIV and HIV-1 infected cells in secondary lymphoid tissue and the multiplicity of infection implied by multiple integration events are consistent with direct movement of virus between contacting cells [16–18].

Several other virus families including rhabdo, herpes, pox, paramyxo, Flavi and African Swine Fever can travel by directed cell-to-cell spread via diverse mechanisms [8]. The induction of virological synapses dictates interaction between the host cell cytoskeleton and the pathogen in ways similar to, but distinct from that described for these other viruses and for intracellular bacteria [19]. Understanding microbial entry and spread reveals a lot about the pathogenesis of the infectious agent, but we can learn as much about host molecular cell biology using pathogens as functional probes, as we can about the pathogens themselves. This will be the case for the virological synapse, which will shed light not only on processes relating to intercellular communication including immunological synapse assembly and function, but may help identify potential molecular targets for intervention in the virus life cycle.

Many of the central questions relating to the cellular and molecular basis of virological synapse structure and function have been, or are being, addressed, and the concept of cell-to-cell spread by these and related structures is well established. Nevertheless, substantial gaps remain in our knowledge, and several of the key concepts relating to this mode of viral spread are controversial and remain to be confirmed or properly understood. This issue of Viruses presents a series of state-of-the art reviews of the field from experts in the major areas of retroviral virological synapse research, discussing areas of particular interest and highlighting significant lacunae in our understanding.
